# Prognostic Significance of Feature-Tracking Right Ventricular Global Longitudinal Strain in Non-ischemic Dilated Cardiomyopathy

**DOI:** 10.3389/fcvm.2021.765274

**Published:** 2021-11-30

**Authors:** Marco Cittar, Alberto Cipriani, Marco Merlo, Giancarlo Vitrella, Marco Masè, Anna Carrer, Giulia Barbati, Manuel Belgrano, Lorenzo Pagnan, Manuel De Lazzari, Benedetta Giorgi, Maria A. Cova, Sabino Iliceto, Cristina Basso, Davide Stolfo, Gianfranco Sinagra, Martina Perazzolo Marra

**Affiliations:** ^1^Cardiovascular Department, Azienda Sanitaria Universitaria Integrata Giuliano Isontina, University of Trieste, Trieste, Italy; ^2^Department of Cardio-Thoraco-Vascular Sciences and Public Health, University of Padua, Padua, Italy; ^3^Biostatistics Unit, Department of Medical Sciences, University of Trieste, Trieste, Italy; ^4^Department of Radiology, Azienda Sanitaria Universitaria Integrata Giuliano Isontina, University of Trieste, Trieste, Italy; ^5^Department of Radiology, Azienda Ospedaliera of Padua, University of Padua, Padua, Italy

**Keywords:** non-ischemic cardiomyopathy, cardiac magnetic resonance feature-tracking analysis, right ventricle global longitudinal strain, prognosis, heart failure

## Abstract

**Aims:** Left ventricular global longitudinal strain (GLS) by cardiac magnetic resonance feature tracking (CMR-FT) analysis has shown an incremental prognostic value compared to classical parameters in non-ischemic dilated cardiomyopathy (NICM). However, less is known about the role of right ventricular (RV) GLS. Our objective was to evaluate the prognostic impact of RV-GLS by CMR-FT analysis in a population of NICM patients.

**Methods:** In this multicenter study, we examined NICM patients evaluated with a comprehensive CMR-FT study. Major cardiac events (MACEs) were considered as the study primary outcome measure and were defined as a composite of (a) cardiovascular death, (b) cardiac transplant or destination therapy ventricular assist device, (c) hospitalization for life-threatening ventricular arrhythmias or implantable cardiac defibrillator appropriate intervention. Heart failure (HF) related events, including hospitalizations and life-threatening arrhythmia-related events were considered as secondary end-points. Receiver operating time-dependent analysis were used to calculate the possible additional effect of RV-GLS to standard evaluation.

**Results:** We consecutively enrolled 273 patients. During a median follow-up of 39 months, 41 patients (15%) experienced MACEs. RV-GLS and LV late gadolinium emerged as the strongest prognostic CMR-FT variables: their association provided an estimated 3-year MACEs rate of 29%. The addition of RV-GLS significantly improved the prognostic accuracy in predicting MACEs with respect to the standard evaluation including LGE (areas under the curve from 0.71 [0.66–0.82] to 0.76 [0.66–0.86], *p* = 0.03). On competing risk analysis, RV-GLS showed a significant ability to reclassify overall both HF-related and life-threatening arrhythmia-related events, regardless of LV and RV ejection fraction.

**Conclusions:** In NICM patients, RV-GLS showed a significant prognostic role in reclassifying the risk of MACEs, incremental with respect to standard evaluation with standard prognostic parameters.

## Background

The implementation of prognostic stratification in non-ischemic dilated cardiomyopathy (NICM) is a demanding issue in clinical practice (1). NICM patients are in fact a specific model of heart failure with reduced ejection fraction, characterized by young patients with low comorbidity profiles and competing risks between heart failure and life-threatening arrhythmias (1). Cardiac magnetic resonance (CMR) is the gold-standard in defining left ventricular (LV) and right ventricular (RV) ejection fraction (EF) as well as tissue characterization, through the late gadolinium enhancement (LGE) assessment (2–4). Feature-tracking (FT) analysis has emerged as a method to study the intrinsic performance of the myocardial wall, able to identify subtle systolic dysfunction. FT-derived LV global longitudinal strain (GLS) assessment has been associated with prognosis in both NICM and ischemic cardiopathy, showing additional prognostic power when combined with the above-mentioned classical parameters (5, 6). However, to date, only few studies have evaluated the prognostic impact of RV-GLS, calculated by CMR-FT analysis, in the setting of NICM (7), despite RVEF is a known prognostic tool in this setting (2). Therefore, the aim of this study was to test the possible prognostic role of RV-GLS measured by FT when added to standard, comprehensive CMR evaluation in a large cohort of Caucasian NICM patients.

## Methods

### Study Population

We retrospectively analyzed all the consecutive patients with a diagnosis of NICM based on current international criteria (8), with an available CMR evaluation, prospectively referred to two Italian Tertiary Referral Centers for the diagnosis and management of cardiomyopathies (Cardiovascular Departments of Trieste and Padua) from July 2008 to August 2017. Inclusion criteria were: LVEF <50% and absence of (a) significant coronary artery disease (stenosis ≥50% of a major coronary artery at coronary angiography or Computed Tomography), (b) significant primary valve disease, (c) congenital heart disease, (d) tachy-induced cardiomyopathy, (e) peripartum cardiomyopathy or (f) acute myocarditis (1, 8).

All the available and readable ECGs were systematically and retrospectively analyzed by three clinicians (i.e., authors MMa, MC, and MMe). The ECG analysis was performed according to the main important acknowledged parameters and measured by standardized measurements (9).

Significant alcohol consumption was defined as ethanol intake >90 g/day for ≥5 years (10). All patients were under evidence-based medical and device treatments (11). This investigation conforms with the principles outlined in the *Declaration of Helsinki* (12) and was approved by the institutional ethical boards of Trieste and Padua Cardiovascular Departments.

### CMR Acquisition Protocol

All patients were assessed as close as possible to the disease onset using 1.5T CMR imaging scanners (Intera, Philips Healthcare, Best, the Netherlands [183 patients]; Magnetom Avanto, Siemens Healthcare, Erlangen, Germany [90 patients]). All cine images were acquired using a balanced, steady-state, free precession (SSFP) sequence during an expiratory breath-hold. Short-axis cine images from cardiac base to apex, and long-axis cine images in 2-, 3-, and 4-chamber views were obtained using the following scan parameters: TE/TR/flip-angle = 1.5 ms/3.0 ms/60°, slice thickness = 8 mm, gap = 2 mm (Intera); TE/TR/flip-angle = 1.0 ms/2.3 ms/60°, slice thickness = 8 mm, gap = 2 mm (Magnetom Avanto). LGE imaging was carried out using a standard LGE technique: two-dimensional segmented breath-held fast low-angle shot inversion recovery sequences (TE/TR/flip-angle = 3 ms/6.1 ms/25°, slice thickness 10 mm, gap = 2 mm [Intera]; TE/TR/flip-angle = 3.2 ms/5.2 ms/25°, slice thickness = 8 mm, gap = 2 mm [Magnetom Avanto]) were applied 10–15 min after contrast agent intravenous administration (gadopentate (Gd-DTPA) or gadobenate dimeglumine (Gd-BOPTA; 0.2 mmol/kg of body weight) in the same views of the cine images; inversion times were adjusted to null normal myocardium using Look-Locker sequence. To exclude artifacts, images were repeated in 2 separate phase-encoding directions.

### CMR Imaging Analysis

All post processing analysis were performed using CVi42® software (Circle Cardiovascular Imaging Inc, Calgary, Canada). Ventricular volumes and systolic function were measured by planimetry of endocardial borders, on short-axis cine images, excluding papillary muscles from the myocardium. LV end-diastolic volume (EDV), LV end-systolic volume (ESV), RVEDV, and RVESV were calculated by summation of these images (“Simpson's rule”). LV mass was calculated by subtracting endocardial from epicardial volume at end-diastole and multiplying by 1.05 g/cm3. Ventricular volumes and LV mass were indexed to body surface area. The LVEF and RVEF were calculated by dividing the stroke volume (EDV minus ESV) by the EDV of the respective ventricle (13). LV focal fibrosis, as demonstrated by LGE, was evaluated and was deemed present only if appreciable on 2 contiguous or orthogonal slices or another readout direction. Patterns of LGE were classified as subendocardial, subepicardial, mid-wall or transmural (14). All measurements were performed by radiologists with ≥10-year experience in cardiac imaging, blinded to patient clinical data.

**Figure 1:**
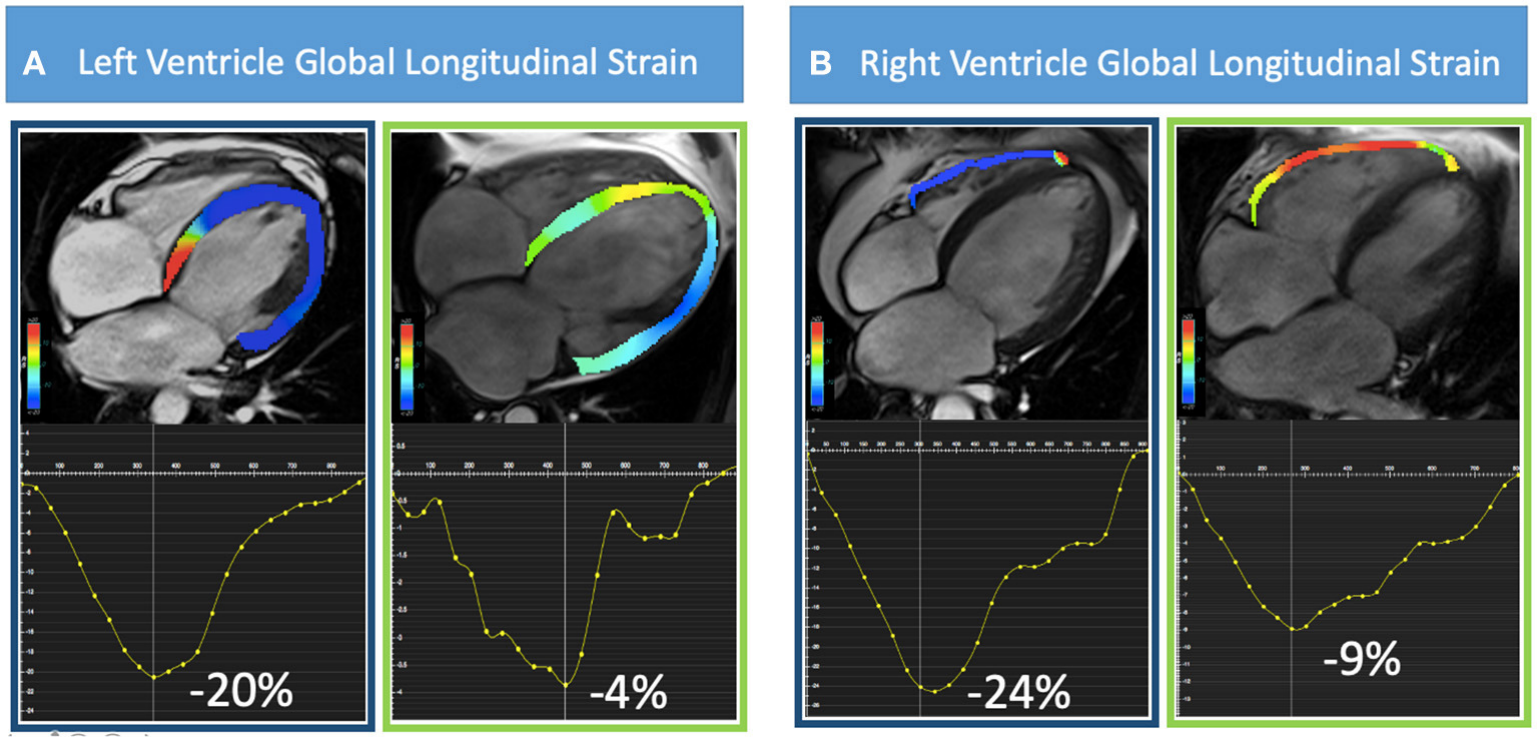
Representative cases of CMR-FT analysis are shown. In **(A)** from left to right, a four-chamber view of patients with preserved (−20%) and reduced (−4%) LV-GLS and in **(B)** preserved (−24%) and reduced (−9%) RV-GLS are illustrated.

For LV short- and long-axis FT analysis, a modified 16-segment LV model derived from the standard American Heart Association 17-segment model was applied omitting the apical cap. An expert operator manually delineated LV endocardial and epicardial borders in all standard cine SSFP short- and long-axis images, with the initial contour set at end-diastole. Values of 2D longitudinal, circumferential and radial peak strain were calculated. For RV strain analysis, we used the 4-chambers view to determine peak global longitudinal strain and 3 short-axis views (basal, mid and apical) for global radial and circumferential strain (Figure 1). Endocardial and epicardial contours were manually drawn during end-diastole with subsequent automatic tracking during the cardiac cycle. Tracking quality was checked using a cine mode, which shows endocardial and epicardial borders tracking throughout the cardiac cycle as well as the resulting strain curves. Segments that did not allow reliable tracking were excluded from analysis. Intercenter reproducibility was measured using a randomly selected sample of 20 cases (10 for each center) by 2 independent observers.

### End-Points

Major cardiovascular events (MACEs) were considered as the study primary outcome measure and were defined as a composite of: (a) cardiovascular death, (b) cardiac transplant or destination therapy ventricular assist device for end-stage heart failure (HF), (c) hospitalization for life-threatening ventricular arrhythmias or implanted cardioverter defibrillator appropriate intervention on sustained ventricular tachycardia >185 beats per minute or ventricular fibrillation (15). Secondary outcomes were: (1) overall cardiovascular mortality; (2) HF-related events defined as a composite of HF death/heart transplant/destination therapy VAD implantation, hospitalization for HF; (3) Arrhythmia-related events: sudden cardiac death or life-threatening ventricular arrhythmias including ICD appropriate intervention. Time to event was calculated as the period between the CMR evaluation and the first MACE. If a single patient experienced more than a single event, the closest event to the CMR study has been used to censor follow-up data. Patients' outcome status was obtained through extensive contact of civic registries, families and general practitioners for patients without recent clinical evaluation. Follow-up ended at the date of end-point or at the last available contact with the patient. No patients included in the study were lost-to-follow-up.

### Statistical Analysis

Continuous variables were expressed as median and interquartile range (IQR) [25°; 75°]. Differences between two groups were compared using Mann-Whitney U test for continuous variables and the chi-square (χ^2^) or Fisher exact test for dichotomous variables, as appropriate. The linear correlation between LVEF-LVGLS, RVEF-RVGLS, LVGLS-RVGLS and LVEF-RVEF was evaluated by means of Pearson's Correlation Coefficient. Kaplan-Meier survival curves and cumulative incidence curves (considering the competing risk of death) were estimated to evaluate the possible association of the considered CMR variables with respect to time to the primary outcome measures and secondary outcome measures, respectively. In the absence of established cut-off values for LV-GLS and RV-GLS at CMR-FT analysis from the literature, median values of our study cohort were used as a cut-off in order to visually compare survival curves and cumulative incidences. Conversely, recognized cut-offs from the literature were used for LVEF and RVEF. Log-rank test and Gray tests were used to assess differences across groups (16). Calculation of hazard ratios (HR) for study outcome measures and 95% confidence intervals (CI) were performed using univariable Cox proportional hazard regression analysis. The HR is calculated for 1-unit increase in the scale of the variable. Given the low number of events, a penalized multivariable Cox model was estimated starting from a list of eight parameters significant at univariable analysis and relevant from a clinical point of view, and the penalized estimation selected the most promising predictors of events (i.e., with a *p*-value < 0.10). Cross-validation was used to choose the optimal value for the tuning parameter lambda1 of the penalized ML estimation. Since it is not possible to estimate the standard errors of the regression coefficients from the penalized estimation (17) a bootstrap-based calculations was performed in order to derive the confidence intervals and p-values reported in Supplementary Table 1. Using this selection, we calculated three additional standard Cox models: Model (a) including clinical variables (i.e., NYHA III/IV class plus presence of sinus rhythm); Model (b) considering clinical variables plus the presence of LGE at CMR; Model (c) including clinical variables, presence of LGE plus RV-GLS considered as a continuous variable. We checked if the proportional hazard assumption in the estimated model was verified by means of the test reported in (18). We also performed an internal validation of the performance estimated model, in terms of calibration and discrimination, by means of a bootstrap procedure (using the function “validate” of the “rms” R package).

Finally, we compared the predictive performance of these models in terms of time-dependent ROC curves, that estimates a AUC suitable for censored data (19). Interobserver and intraobserver variability were analyzed using the intraclass correlation coefficient (ICC) on a group of 46 subjects (four measures per subject: two different operators and for each operator a double measurement on each subject at least 1 day in blind mode). This allowed us to achieve 80% power to detect an ICC of 0.90 under the null hypothesis of ICC = 0.80, by using an F-test at a significance level of 0.05 (20) (Supplementary Table 2), finally the method used to calculate ICC is a mixed effects model (i.e., when patients effects are treated as random and the raters effects are treated as fixed) evaluating the absolute agreement between raters. A *p*-value of <0.05 was considered statistically significant, except in the univariable covariate's selection as explained above. Analyses were performed using IBM SPSS Statistical Package 20 (IBM, Armonk, New York) and R statistical software version 3.3.2 (R Foundation for Statistical Computing, Vienna, Austria), libraries “cmprsk,” “coxphf” and “timeROC.”

## Results

### Study Population

Study population counted 273 patients (men 66%, median age 51; LVEF 34%; LGE present in 52%; median difference from disease onset to CMR: 1 month [IQR 0–3 months]) followed for a median follow-up of 39 months (IQR 20-71). During follow-up, 49 MACEs occurred: 10 cardiovascular deaths (nine due to HF and one sudden cardiac death), 16 cardiac transplants, four VAD implantations, 15 appropriate ICD interventions, and four hospitalizations due to life-threatening arrhythmias. Because only the first event was censored, 41 MACEs (15%, 5/100 patients/year) were eligible for statistical analysis. No other cause of death other than cardiovascular were found in the present population. Finally, 44 patients were hospitalized due to HF during follow-up. Tables 1, 2 summarize baseline clinical, demographic, therapeutic and CMR-FT characteristics of patients with and without MACEs. Compared to survivors, patients with MACEs showed more frequently NYHA classes III-IV and less frequently sinus rhythm. Moreover, at CMR evaluation, they presented a significantly reduced EF of both ventricles and more frequently displayed LGE. Finally, they had significantly more impaired LV- and RV-GLS (LV-GLS −8% vs. −11.3%, *p* = 0.001; RV-GLS −15.8% vs. −20%, *p* = <0.001 respectively. Finally, we found moderate to strong correlations between the major CMR variables (Supplementary Figure 1).

**Table 1:** Characteristics of the study population according to experience of the primary end-point^*^.

	**All patients** **(*n* = 273)**	**Patients with MACEs** **(*n* = 41)**	**Patients without MACEs** **(*n* = 232)**	***p***
**Clinical data**				
Male sex	181 (66%)	28 (68%)	153 (66%)	0.460
Age, yrs	51 [41; 60]	46 [35; 69]	51 [41; 60]	0.110
NYHA Class III/IV	64 (23%)	22 (54%)	42 (18%)	**<0.0001**
Atrial fibrillation	25 (8%)	9 (22%)	16 (6%)	**0.002**
LBBB, ECG	55 (21%)	7 (17%)	48 (21%)	0.360
LV Hypertrophy, ECG	70 (26%)	9 (22%)	61 (27%)	0.327
**Comorbility**				
Hypertension	95 (35%)	13 (32%)	82 (25%)	0.397
Diabetes/IGT	43 (16%)	7 (17%)	36 (16%)	0.477
Familial cardiomiopathy	55 (20%)	11 (27%)	44 (19%)	0.174
Alcohol abuse	23 (8%)	1 (2%)	22 (10%)	0.109
Chronic renal failure	20 (7%)	5 (12%)	15 (7%)	0.163
**Laboratory data**				
BUN, mg/dL	30 [18; 41]	33 [21; 40]	28 [17; 41]	0.459
Serum creatinine, mg/dL	0.95 [0.8; 1.12]	0.97 [0.76; 1.19]	0.95 [0.8; 1.1]	0.559
Hb, g/dL	13.8 [12.7; 14.9]	13.8 [12.7; 14.5]	13.9 [12.7; 15]	0.392
**Therapy**				
β-blockers	250 (92%)	37 (90%)	213 (92%)	0.465
ACEi/ARBs/ARNi	252 (92%)	38 (93%)	214 (92%)	0.610
MRA	129 (47%)	27 (66%)	102 (44%)	**0.008**

*Values are median [IQR] for continuous variable or n (%) in binary variables*.

*ACEi, angiotensin converter enzyme inhibitors; ARBs, angiotensin receptor blockers; ARNi, Angiotensin Receptor Neprilysin Inhibitor; BUN, blood urea nitrogen; GLS, global longitudinal strain; IGT, impaired glucose tolerance; LBBB, left bundle branch block; LGE, late gadolinium enhancement; LV, left ventricular; LVEF, left ventricle ejection fraction; MACEs, major cardiovascular events; MR, mitral regurgitation; MRA, mineralocorticoid receptor antagonists; NYHA, New York Heart Association; RV, right ventricle; RVEF, right ventricle ejection fraction*.

*Missing values: BUN 28%, Serum creatinine 12%, Hb 14%. No-missing values for other parameters*.

* *MACEs were considered as the study primary outcome measure and were defined as a composite of: (a) cardiovascular death, (b) cardiac transplant or destination therapy ventricular assist device for end-stage heart failure (HF), (c) hospitalization for life-threatening ventricular arrhythmias or implanted cardioverter defibrillator appropriate intervention on sustained ventricular tachycardia >185 beats per minute or ventricular fibrillation*.

*Bold values correspond to significative p of interaction (p-value < 0.05)*.

**Table 2:** Baseline CMR-FT parameters of the study population according to experience of the primary end-point^*^.

	**All patients** **(*n* = 273)**	**Patients with MACEs** **(*n* = 41)**	**Patients without MACEs** **(*n* = 232)**	***p***
**Standard CMR-data**				
LVEDVi, ml/m2	125 [107; 159]	162 [120; 180]	123 [104; 153]	**<0.0001**
LVEF, %	34 [25; 43]	25 [21; 33]	36 [27; 44]	**<0.0001**
RVEF, %	51 [40; 59]	37 [33; 52]	53 [44; 60]	**<0.0001**
LGE presence	140 (52%)	31 (76%)	109 (48%)	**0.001**
**CMR-FT strain values**				
LV peak GRS, %	20.4 [12.7; 27.1]	13.7 [8.2; 21]	22 [14; 27.4]	**0.002**
LV peak GCR, %	−10.7 [−7.8; −13.5]	−8.2 [−5.7; −11.3]	−11.3 [−8; −13.6]	**0.003**
LV peak GLS, %	−10.7 [−7; −13.7]	−8 [−6.6; −10.7]	−11.3 [−7.7; 13.9]	**0.001**
RV peak GRS, %	17.6 [12; 23.7]	14.6 [9.9; 20.7]	18.5 [12.9; 24.1]	**0.006**
RV peak GCS, %	−10.5 [−7.5; −13.2]	−8.8 [−5.4; −11.2]	−10.8 [−7.7; −13.3]	**0.013**
RV peak GLS, %	−19.1 [−15.4; −23]	−15.8 [−11.4; −18.6]	−20 [−16.4; −23.8]	**<0.0001**

*Values are median [IQR] for continuous variable or n (%) in binary variables*.

*CMR, cardiac magnetic resonance; FT, feature tracking; GCS, global circumferential strain; GLS, global longitudinal strain; GRS, global radial strain; LGE, late gadolinium enhancement; LV, left ventricle; LVEDVi, left ventricle end diastolic volume indexed; LVEF, left ventricle ejection fraction; MACEs, major cardiovascular events; RV, right ventricle; RVEF, right ventricle ejection fraction*.

*No missing values were present*.

* *MACEs were considered as the study primary outcome measure and were defined as a composite of: (a) cardiovascular death, (b) cardiac transplant or destination therapy ventricular assist device for end-stage heart failure (HF), (c) hospitalization for life-threatening ventricular arrhythmias or implanted cardioverter defibrillator appropriate intervention on sustained ventricular tachycardia >185 beats per minute or ventricular fibrillation*.

*Bold values correspond to significative p of interaction (p-value < 0.05)*.

### The Prognostic Role of RV-GLS

At CMR-FT evaluation, we found that LGE, LV end-diastolic volume, LVEF, RVEF, LV-GLS and RV-GLS were all associated to MACEs, as shown at univariable analysis (Table 3). Multivariable analyses, derived from the penalized model including the variables shown in Supplementary Table 1, showed RV-GLS as independently associated to MACEs, along with LGE, NYHA classes III-IV and sinus rhythm (Table 3).

**Table 3:** CMR-FT model. Uni- and multivariable Cox analysis to predict MACEs (primary end-point)^*^.

	**Univariable analysis** **HR (95% CI)**	***p***	**Multivariable analysis** **HR (95% CI)**	***p***
**Clinical data**				
NYHA III-IV	3.97 (2.15–7.33)	<0.0001	2.98 (1.60–5.55)	0.001
Sinus rhythm	0.25 (0.12–0.53)	<0.0001	0.35 (0.17–0.75)	0.007
**Standard CMR-data**				
LVEDVi, ml/m2	1.01 (1–1.02)	<0.0001		
RVEDVi, ml/m2	1.02 (1.01–1.03)	<0.0001		
LVEF, %	1.08 (1.04–1.11)	<0.0001		
RVEF, %	1.05 (1.03–1.08)	<0.0001		
LGE presence	3.14 (1.54–6.4)	0.002	2.51 (1.22–5.13)	0.012
**CMR-FT strain values**				
LV peak GLS, %	1.08 (1.02–1.15)	0.008		
RV peak GLS, %	1.06 (1.02–1.1)	0.001	1.06 (1.02–1.1)	0.008

*CI, confidence interval; CMR, cardiac magnetic resonance; FT, feature tracking; GCS, global circumferential strain; GLS, global longitudinal strain; GRS, global radial strain; HR, hazard ratio; LGE, late gadolinium enhancement; LV, left ventricle; LVEDV, left ventricle end diastolic volume indexed; LVEF, left ventricle ejection fraction; RV, right ventricle; RVEF, right ventricle ejection fraction*.

*Only significant variables were reported in univariable analysis*.

* *MACEs were considered as the study primary outcome measure and were defined as a composite of: (a) cardiovascular death, (b) cardiac transplant or destination therapy ventricular assist device for end-stage heart failure (HF), (c) hospitalization for life-threatening ventricular arrhythmias or implanted cardioverter defibrillator appropriate intervention on sustained ventricular tachycardia >185 beats per minute or ventricular fibrillation*.

**Figure 2:**
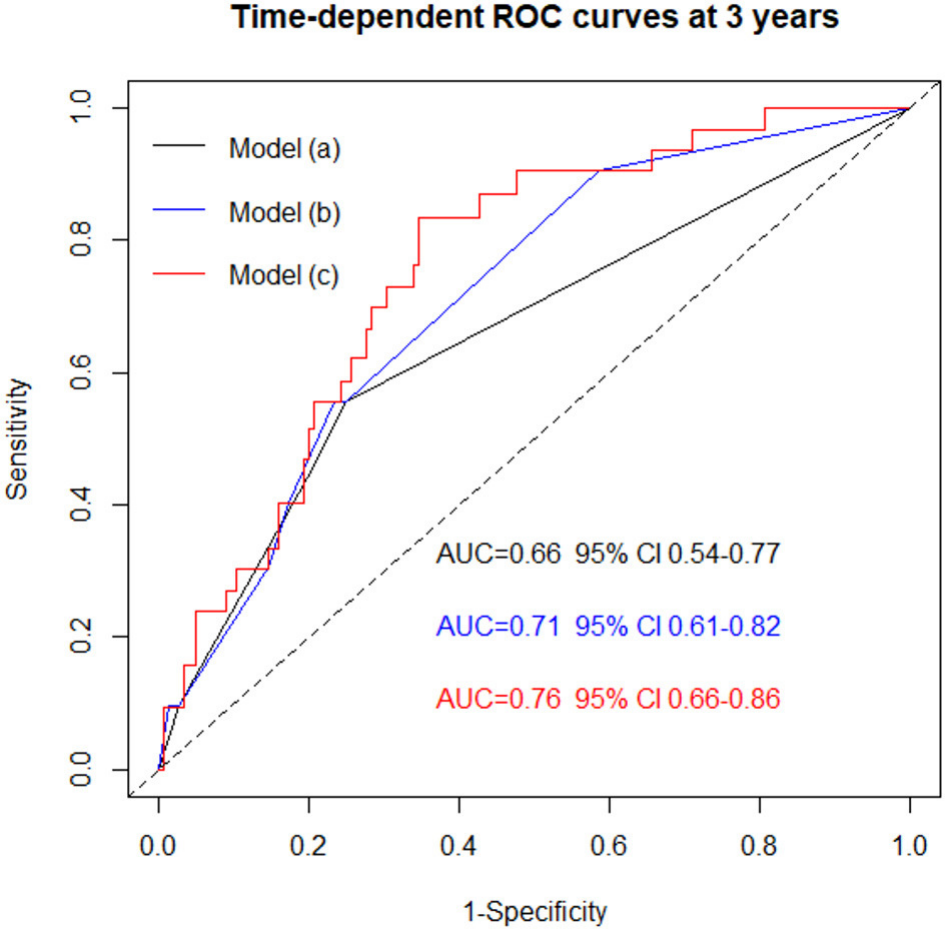
Time dependent ROC curves showing the progressive incremental power of CMR analysis in predicting MACEs when adding LGE (model b) and LGE + RV-GLS (model c) to clinical model (i.e., model a: NYHA III-IV + sinus rhythm). The three models are derived from the multivariable analysis is showed in Table 3. Model a vs. Model b, *p* = 0.03. Model a vs. Model c, *p* = 0.01. Model b vs. Model c, *p* = 0.03. AUC, area under the curves; CMR, cardiac magnetic resonance; LGE, late gadolinium enhancement; MACEs, major cardiovascular events; NYHA, New York heart association; ROC, receiver operating curves; RV-GLS, right ventricular global longitudinal strain.

Receiver operating time-dependent analysis derived from the multivariable models, showed a progressively incremental prognostic role of CMR variables in predicting MACEs: model (a), the “clinical model,” showed the prognostic power of NYHA class III-IV and sinus rhythm (AUC of 0.66 [0.54–0.77]; model (b), the “clinical model” plus LGE, increased the AUC to 0.71 (0.61–0.82), *p* = 0.03 vs. model (a); model (c), including model (b) plus the RV-GLS as a continuous variable, further increased the AUC to 0.76 (0.66–0.86), *p* = 0.03 vs. model (b) (Figure 2).

**Figure 3:**
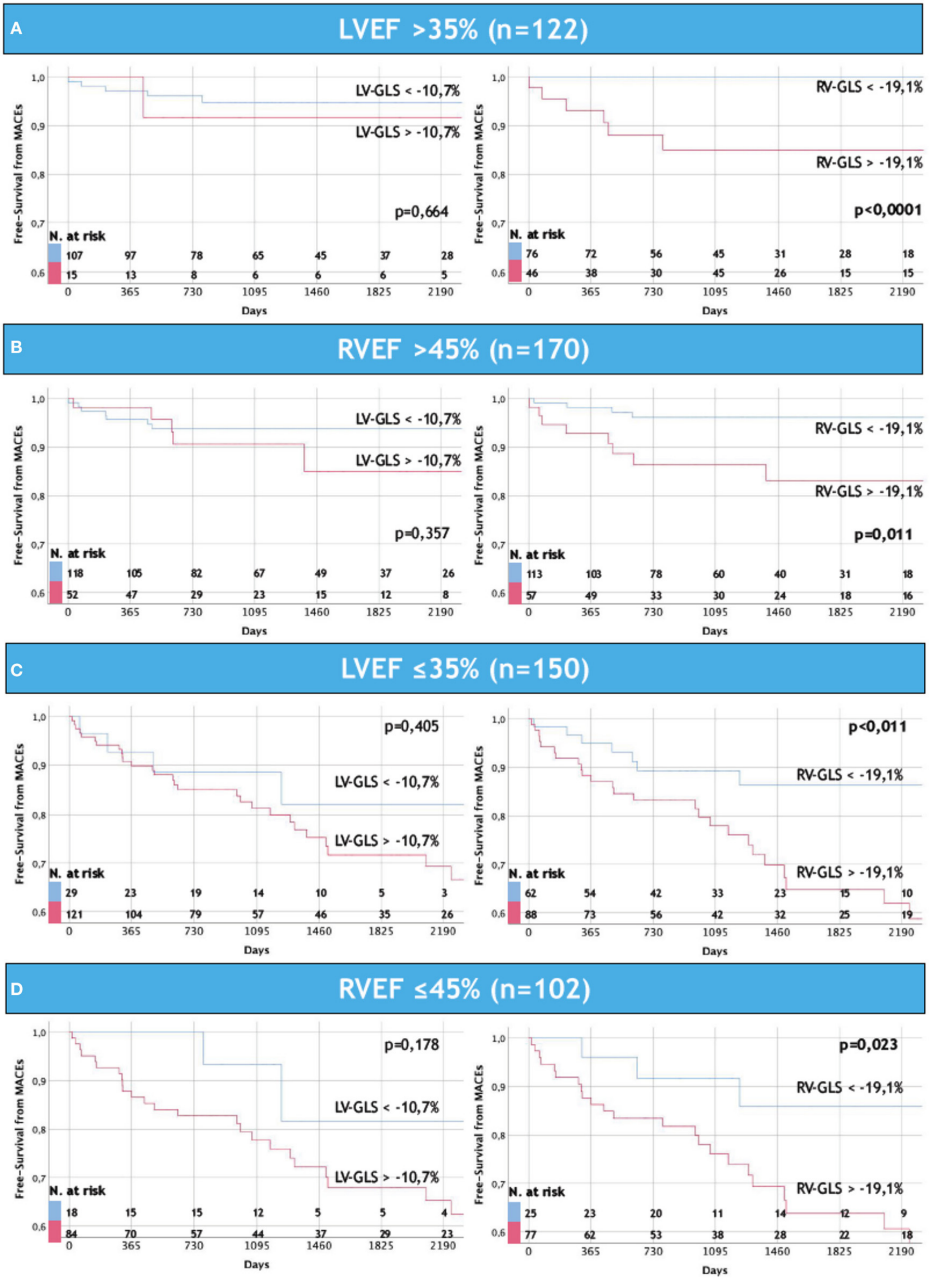
Ability of RV-GLS to further stratify MACEs in NICM patients regardless severe LV and RV dysfunctions. Note how RV-GLS identifies MACEs independently to EF: in **(A,B)** are depicted patients with non-severe reduction of left **(A)** and right **(B)** ventricular ejection fraction, while in **(C,D)** are shown the remaining patients with severe reduction of left **(C)** and right **(D)** ventricular ejection fraction. The same power is not appreciated by LV-GLS in this recently onset NICM population. LVEF, left ventricular ejection fraction; LV-GLS, left ventricular global longitudinal strain; RVEF, right ventricular ejection fraction; RV-GLS, right ventricular global longitudinal strain; MACEs, major cardiovascular events.

Consistently with multivariable analysis, RV-GLS >-19.1% (i.e., the median value found in our population) was significantly associated to higher rates of MACEs, independently to RVEF and LVEF. Of note, LV-GLS did not show the same prognostic value (Figure 3).

**Figure 4:**
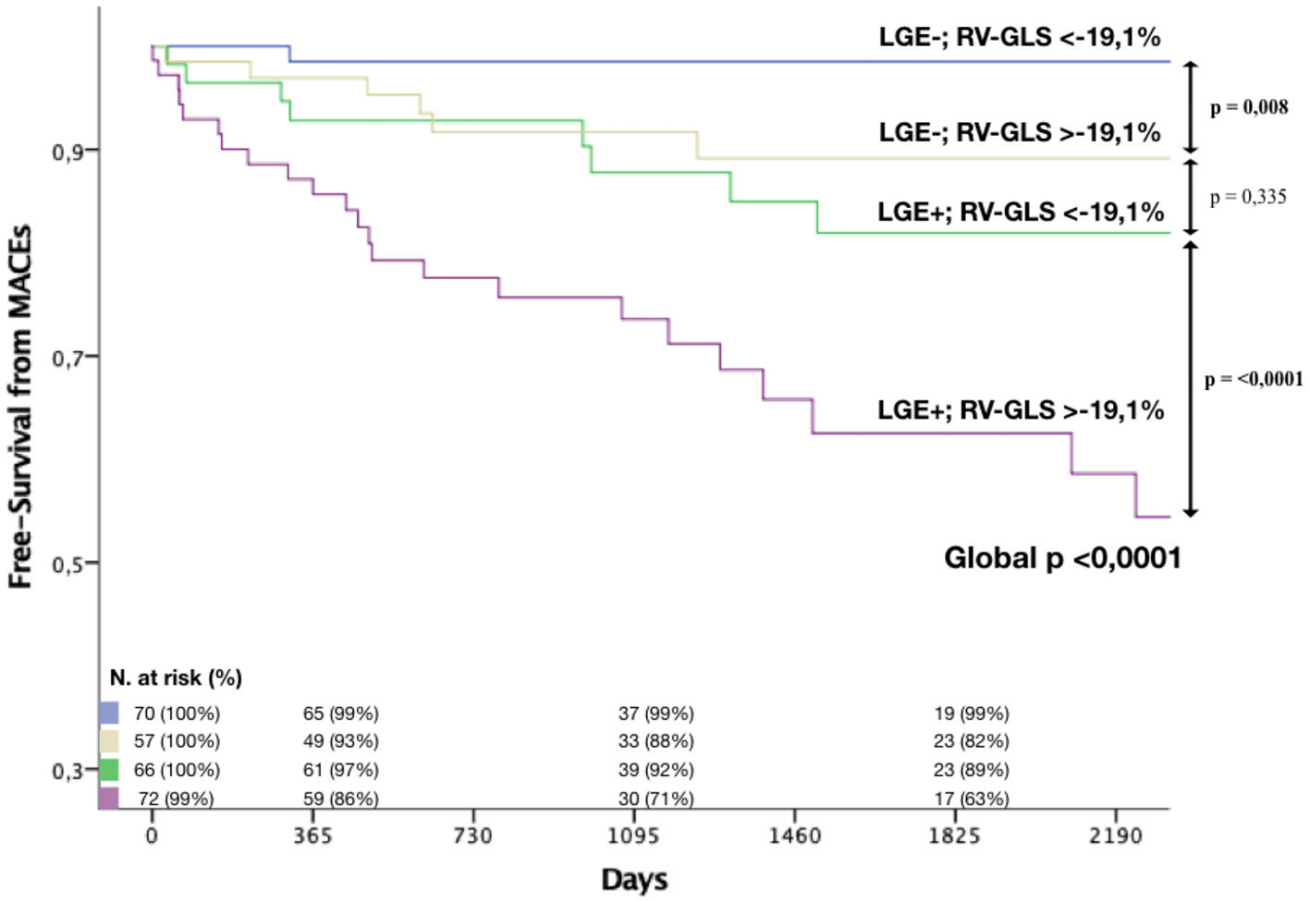
Kaplan-Meier curves. The association of RV-GLS > −19% and LGE were strongly associated to MACEs in NICM patients. Blue curve shows survival in patients without LGE and with preserved RV-GLS; yellow curve shows survival in patients without LGE and with reduced RV-GLS; green curve shows survival in patients with LGE and preserved LV-GLS; purple curve shows survival in patients with LGE and reduced RV-GLS. LGE, late gadolinium enhancement; RV-GLS, right ventricular global longitudinal strain; MACEs, major cardiovascular events.

The simultaneous presence of LGE and RV-GLS >-19.1% was associated to particularly poor outcomes (estimated 3-year and 5-year MACEs rate of 29% and 37% respectively). On the contrary, patients without LGE and with preserved RV-GLS (<-19.1%) showed. a very good prognosis, with an estimated 3-year and 5-year MACEs rate of 1% (Figure 4).

**Figure 5:**
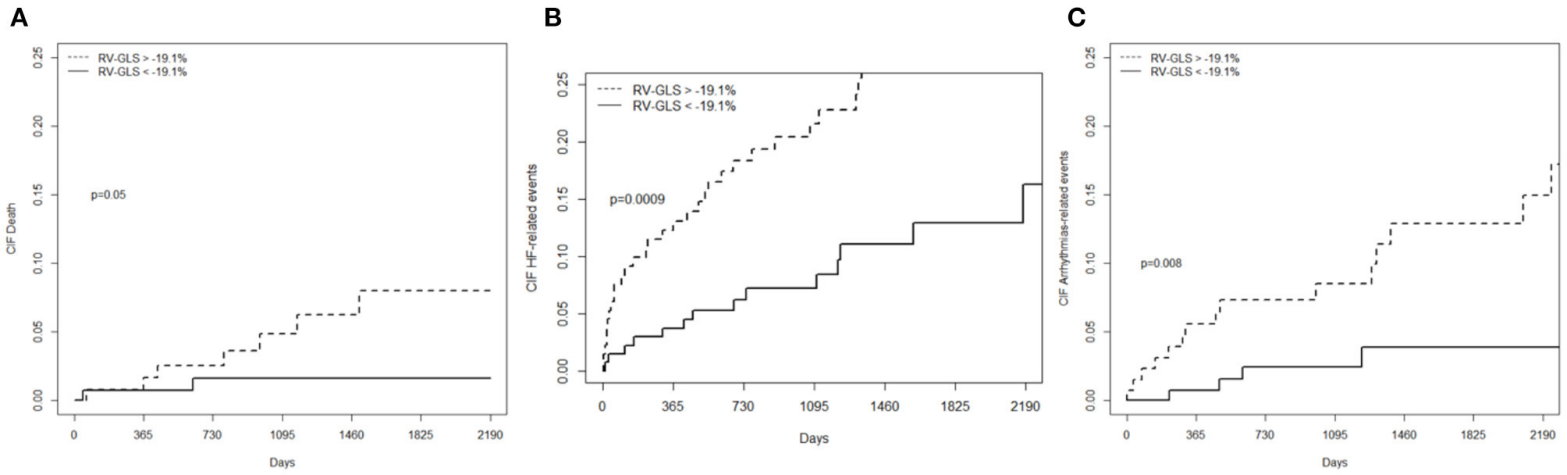
Cumulative incidence curves showing the significant association between RV-GLS and secondary endpoints: **(A)** overall cardiovascular mortality; **(B)** HF-related events (HF death/heart transplant/destination therapy VAD implantation, hospitalization for HF); **(C)** Life threatening arrhythmia-related events (sudden cardiac death or life-threatening ventricular arrhythmias including ICD appropriate intervention). RV-GLS confirms its ability to predict events also in secondary endpoints. CIF, cumulative incidence curves; MACEs, major cardiovascular events; RV-GLS, right ventricular global longitudinal strain.

**Figure 6:**
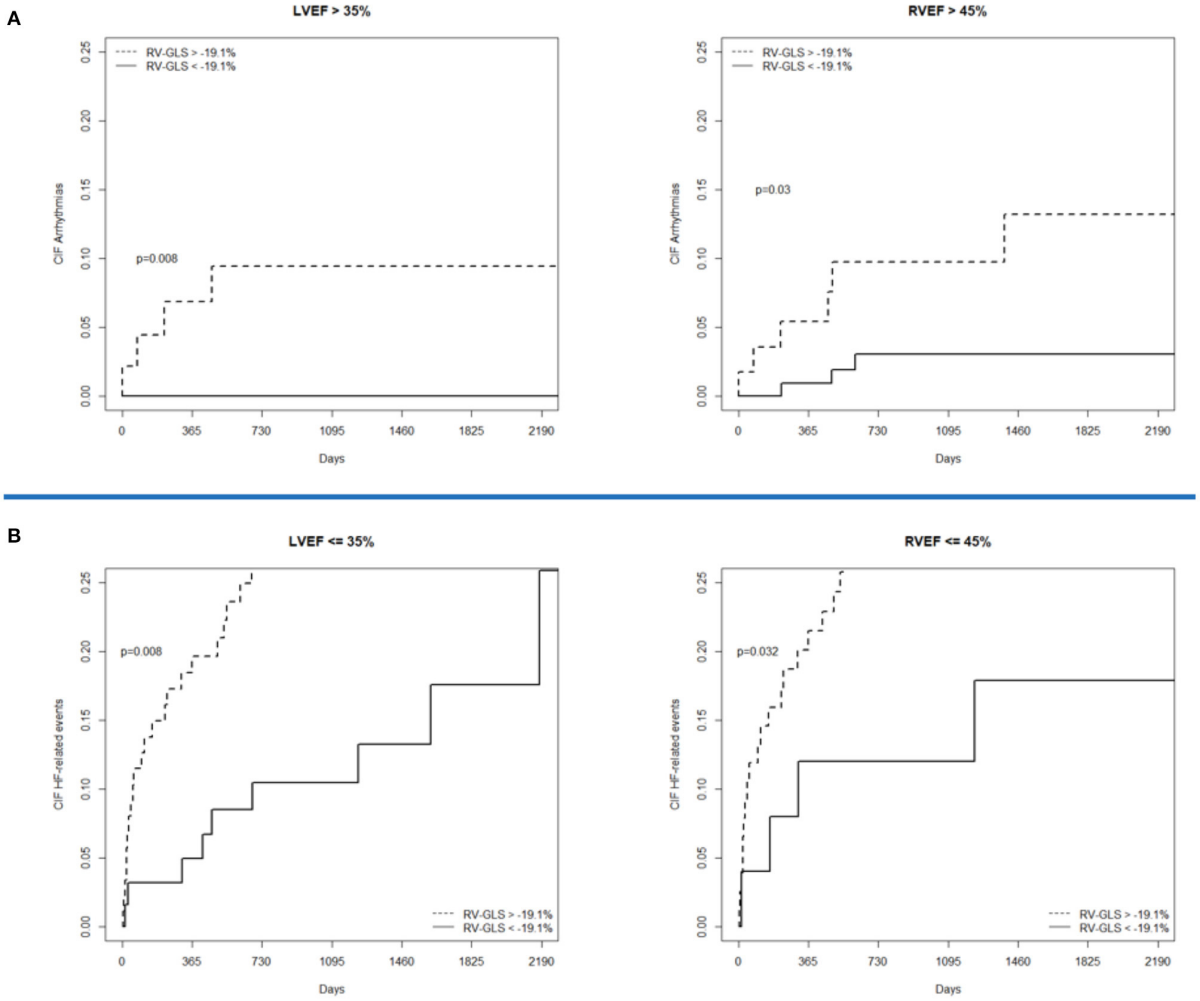
Cumulative incidence curves showing the association of RV-GLS in secondary endpoints such as life-threatening arrhythmia-related events and HF-related events after stratification for LVEF and RVEF. In **(A)** RV-GLS discriminates patients at risk of arrhythmic events in those with LVEF and RVEF are not severely depressed whereas, in **(B)** discriminates patients at risk of HF related events (including HF hospitalizations) in those with severe reduction of EF, both left and right. HF, heart failure; LVEF, left ventricular ejection fraction; RVEF, right ventricular ejection fraction; RV-GLS, right ventricular global longitudinal strain.

Concerning secondary outcomes measures, RV-GLS >-19.1% was associated to higher rates of cardiovascular mortality, HF-related events, and life-threatening arrhythmia-related events. Therefore, RV-GLS was capable of predicting also individual components of MACEs (Figure 5). Moreover, RV-GLS showed ability to reclassify arrhythmia-related events mostly in patients with LVEF >35% and RVEF >45% and HF-related events mostly in patients with LVEF <35% and RVEF <45% (Figure 6). Finally, RV-GLS >-19.1% was associated to higher rates of HF-related events (both excluding HF hospitalization and considering HF hospitalization alone) particularly in patients with LVEF <35% and RVEF <45% (Supplementary Figure 2).

### Interobserver and Intraobserver Variability

All the ICC for CMR-FT measurements and RVEF are reported in Supplementary Table 2. ICC for RV-GLS of intraobserver repeatability was 0.91 (95% CI 0.83–0.95) while ICC of interobserver repeatability was 0.88 (95% CI 0.79–0.93). ICC for RVEF of intraobserver repeatability was 0.95 (0.92–0.96) while ICC of interobserver repeatability was 0.92 (0.85–0.94).

## Discussion

The present study shows for the first time an independent prognostic role of FT-derived RV-GLS, when added to standard clinical parameters and comprehensive CMR evaluation, in a large cohort of Caucasian NICM patients: in Figure 2, it is evident that patients presenting with NYHA classes III-IV, no sinus rhythm, LGE and reduced RV-GLS, are at a significantly increased risk of developing MACEs.

Our population included “recently onset NICM patients” (i.e., 1 month) and allowed us to explore the possible prognostic impact of RV function (and in particular RV-GLS) over the LV function in the initial, crucial phases of the medical treatment in NICM patients. In fact, RV improvements under therapy might be faster than LV reverse remodeling, as previously suggested (21), and might emerge as an early therapeutic and prognostic target.

The prognostic impact of RV-GLS emerged in predicting the MACEs, independently from LVEF, RVEF and respect its counterpart LV-GLS (Figure 3), and both HF-related and life-threatening arrhythmia-related events (Figure 5). Finally, on exploratory analysis, RV-GLS appears as a potential additional prognostic tool in the arrhythmic stratification of patients without severe LV and RV dysfunctions, and in the HF-related stratification of patients with severe LV and RV dysfunctions. In those challenging subgroups, RV-GLS might potentially identify patients who might benefit from closer clinical evaluations (Figure 6; Supplementary Figure 2).

Compared to standard clinical and CMR features, the possible additive significance of RV strain was widely unexplored, despite RV dysfunction is a known prognostic tool in NICM, when measured by RVEF (2). Despite previous reports addressed the clinical utility of RV-GLS assessment at speckle tracking echocardiography evaluation in the broad setting of heart failure with reduced ejection fraction (22), few data existed about FT-derived RV-GLS in the specific NICM setting. So far, data on the role of RV-GLS in the setting of NICM were available only in highly selected cohorts (Asian, without AF, HF in stages C and D). Conversely, our data on a large Caucasian NICM population, highlighting the prognostic role of a comprehensive evaluation of biventricular function through standard and emerging CMR techniques, appear novel, reliable and potentially impactful in clinical management of those patients (7). The present results appear clinically relevant and potentially useful in the global assessment of challenging patients such as those affected by NICM. It's well-known that echocardiography is the first choice method to study systolic and diastolic function, due to his wide availability and reproducibility. Recent data suggested a possible link between RV-GLS measured with speckle tracking echocardiography and adverse outcome in NICM (23, 24). However, it is also known that RV evaluation in echocardiography can suffer from limitations such as poor acoustic window and RV anatomical position (25). Furthermore, so far the amount of data about CMR-FT analysis in NICM were mostly focused on LV strain analysis (5, 6).

### The Vertical Ventricle

RV is a crescent-shaped structure. Traditionally, RV is divided in 3 anatomical regions: (1) inlet; (2) apex; (3) outlet. The thin RV free wall is histologically arranged in two main layers, the superficial (with circumferentially oriented myocytes) and a more represented subendocardial sheet (with longitudinally oriented fibers). Physiologically, RV function is preload-based. It guarantees a nearly constant stroke volume streamlining blood flow in a low impedance circulation. RV stroke volume is generated by the coupling with the LV (20–40%) and by intrinsic RV contraction which, probably due to the predominantly longitudinal architecture, is mainly developed by vertical shortening (26). This assumption might explain the tight relationship between RV-GLS and outcomes in NICM patients.

Many contributors may lead to RV dysfunction (RVD) in NICM: (1) LV dysfunction; (2) pressure overload due to pulmonary hypertension; (3) mitral regurgitation; (4) the cardiomyopathic process itself. On the other hand, given the strict ventricular interdependence, RVD might further impair LV function thereby aggravating prognosis of NICM patients (27, 28).

### RV-GLS: Relationship With RVEF

Despite CMR is recognized as the gold standard technique for RV systolic function assessment, tissue deformation analysis might identify subtle RV dysfunction, undetectable by RVEF (29). This has been suggested at echocardiographic analysis (30) but has never been described in a CMR study on a large NICM population. The results of this study show how RV-GLS should be integrated in the CMR evaluation, implementing the prognostic information obtained by RVEF measurement. Future studies will be necessary to confirm the cut-off value here suggested of −19,1%, derived from the median value in our population, in the absence of referral values in literature.

### RV-GLS: Relationship With LV Function

RV-GLS was demonstrated to be a prognostic feature in our NICM population, independently from LV function, measured by both LVEF and LV-GLS (28, 31). These results could be explained by the relatively short follow-up time (3 years). As a matter of fact, it is well-known that the prognostic power of LV systolic function in NICM patients is more evident in the long-term (32). As a consequence, it clearly emerges the necessity of an early global evaluation of NICM patients, which should include a systematic comprehensive CMR morpho-functional and deformation biventricular assessment other than tissue characterization, in order to provide a more complete prognostic stratification, particularly in the short-term. Furthermore, it could be speculated that RV-GLS may be a helpful tool for better selection of candidates to ICD in patients with non-severe LV dysfunction and, on the other hand, to LV assist device or for better estimating the timing for heart transplant in patients with severe LV or RV dysfunction (Figure 6). Nevertheless, even if interesting, further studies are needed to confirm these exploratory findings, that should be only hypothesis-generators.

### RV-GLS: Relationship With LGE

Given its ability to detect myocardial scar tissue, the presence of LGE is currently recognized as the most powerful CMR prognostic finding in NICM (3, 4, 33). From our results, after including RV-GLS in the CMR-FT evaluation, a significant increase in AUC was reached in comparison not only to standard clinical evaluation but also to LGE (Figure 2). The presence of a reduced RV-GLS associated to the presence of LGE identified the highest-risk patients with a MACEs estimated risk of 29% at 3 years (Figure 4). Therefore, an impaired RV-GLS appears to confer a higher risk of events, independently to the presence of LGE, which is one of the strongest predictors in NICM (33). This finding might be explained by the fact that both ventricles are affected from the cardiomyopathic process.

### Study Limitations

This study suffers by the common referral and inclusion biases of retrospective observational studies. Despite the study population is the largest NICM population in which RV-GLS prognostic impact has been evaluated during an adequate follow-up period, the present results cannot be generalized to all NICM patients. Furthermore, an external validation is cohort might be required to confirm the hypothesis generated by our model regarding the use of RV-GLS in clinical practice. The results of internal validation (i.e., a moderate rate of optimism in the calibration slope and in discrimination evaluated by means of a boostrap procedure, respectively 0.09 and 0.02) only partially overcome this limit. To date, Feature Tracking RV GLS is not validated because of the lack of large studies based on this method. We assessed reproducibility between high-trained expert in cardiovascular imaging and the results were consistent and reliable. However in future, large studies are needed to confirm these data and to compare CMR data to echocardiographic data. Important variables were not routinely performed, with a high rate of missing values, especially regarding NTproBNP, which could not be used for analysis. Also, some CMR data were not systematically available in both centers, such as both atrial volumes. Although CMR is crucial for NICM assessment, it was not performed in all the patients who eventually received a diagnosis of NICM in the two centers involved, especially in the first years of enrollment period. This, however, is a real-world limitation and the present results highlight how the availability of this methodic should be further implemented. LGE has been treated as a categorical variable since its quantification is not definitely validated in literature. Despite the penalized multivariable procedure adopted, multivariable analysis results should be used as hypothesis generating, due to the limited number of events. Furthermore, due to the retrospective nature of the study, multivariable analysis has not been performed for secondary endpoints. We acknowledge that ICD appropriate interventions do not always correspond to SCD, however they have been considered in the MACEs and in the arrhythmia-related end-points due to the relevance of this event in the natural history of the disease, as previously reported (34). Even though low event-rate is a known limitation of studies on NICM (35) and our population represents, to the best of our knowledge, the largest existing NICM group evaluated with a complete biventricular CMR-FT assessment, higher number of events are needed in order to build more comprehensive and multi-parametric multivariable models. This should be accomplished by larger, possibly prospective studies.

## Conclusions

In recently-onset NICM patients, FT-derived RV-GLS impairment emerges as strongly associated with MACEs. Given this, RV-GLS appears to be a promising tool able to further re-classify patient's risk independently from LVEF, RVEF and LV-GLS and potentially incremental if compared to LGE. Furthermore, RV-GLS might be a tool for implementing the prediction of arrhythmia- and HF-related events in patients with LVEF >35% and of HF-related events in patients with LVEF <35%. In conclusion, a comprehensive CMR-FT study, always complementary to an advanced systolic and diastolic echocardiographic evaluation, should be systematically performed in patients with NICM, including RV-GLS, in order to globally improve the prognostic stratification and therapeutic management of this population.

## Data Availability Statement

The raw data supporting the conclusions of this article will be made available by the authors, without undue reservation.

## Ethics Statement

The studies involving human participants were reviewed and approved by the Institutional Ethical Boards of Trieste and Padua Cardiovascular Departments. The patients/participants provided their written informed consent to participate in this study.

## Author Contributions

MC: conception and design of paper, analysis and interpretation of data, drafting of the manuscript, and critical revision of the manuscript. ACi and MMe: design of the analysis and interpretation of data, drafting of the manuscript, and critical revision of the manuscript. GB: statistical analysis. GV, MMa, MB, LP, MAC, ACa, MD, and BG: analysis and interpretation of data and drafting of the manuscript. CB, DS, and SI: critical revision of the manuscript. GS and MP: drafting of the manuscript and critical revision of the manuscript. All authors contributed to the article and approved the submitted version.

## Conflict of Interest

The authors declare that the research was conducted in the absence of any commercial or financial relationships that could be construed as a potential conflict of interest.

## Publisher's Note

All claims expressed in this article are solely those of the authors and do not necessarily represent those of their affiliated organizations, or those of the publisher, the editors and the reviewers. Any product that may be evaluated in this article, or claim that may be made by its manufacturer, is not guaranteed or endorsed by the publisher.

## Abbreviations

AUCs: area under the curves
CI: confidence interval
CMR: cardiac magnetic resonance
CRT: cardiac resynchronization therapy
EDV: end diastolic volume
ESV: end systolic volume
FT: feature tracking
GLS: global longitudinal strain
HF: Heart Failure
HR: hazard ratio
ICC: intraclass correlation coefficient
ICD: implantable cardiac defibrillator
IQR: interquartile range
LGE: late gadolinium enhancement
LV: left ventricle
LVEF: left ventricle ejection fraction
LVRR: left ventricle reverse remodeling
MACEs: Major Cardiac Events
NICM: non-ischemic dilated cardiomyopathy
ROC: receiver operating characteristics
RV: right ventricle
RVD: Right ventricular dysfunction
RV-GLS: right ventricle global longitudinal strain
RVEF: right ventricle ejection fraction
SSFP: steady-state free precession
STE: speckle tracking echocardiography
SVT: sustained ventricular tachycardia
VF: ventricular fibrillation
χ^2^: chi-square.
