# Supplementary figures and images for: Prognostic Significance of Feature-Tracking Right Ventricular Global Longitudinal Strain in Non-ischemic Dilated Cardiomyopathy
Source: Front Cardiovasc Med. 2021 Nov 30;8:765274. doi: 10.3389/fcvm.2021.765274 (PMC8669391; doi:10.3389/fcvm.2021.765274)

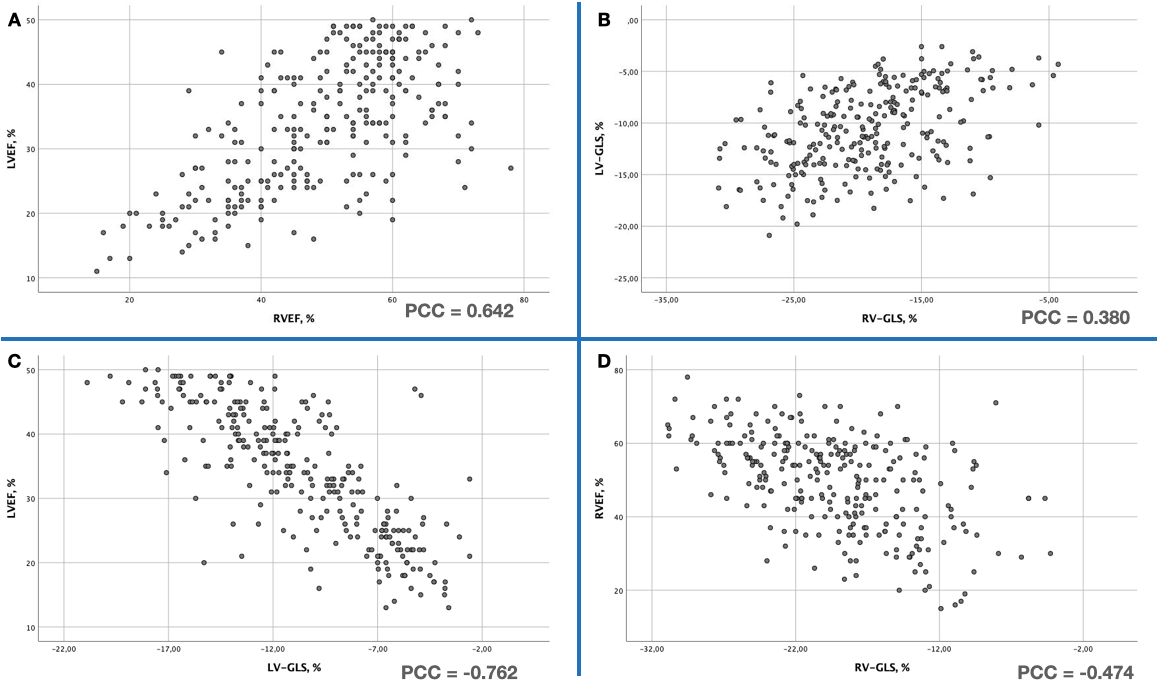

Supplement: Supplementary file 1 [file Image_1.tiff]

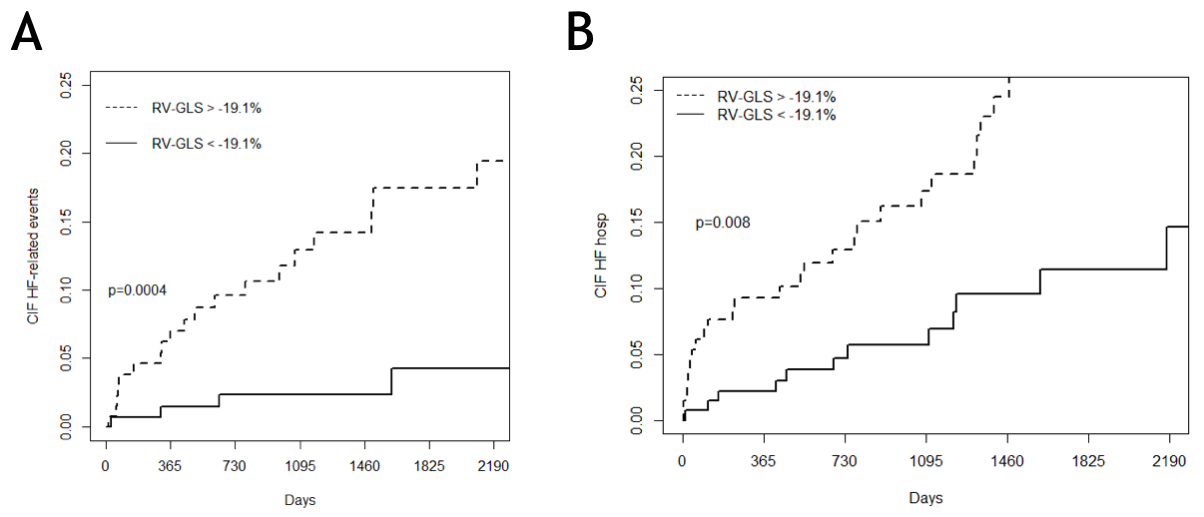

Supplement: Supplementary file 2 [file Image_2.tiff]
