# Supplementary material for: Prognostic Significance of Feature-Tracking Right Ventricular Global Longitudinal Strain in Non-ischemic Dilated Cardiomyopathy
Source: Front Cardiovasc Med. 2021 Nov 30;8:765274. doi: 10.3389/fcvm.2021.765274 (PMC8669391; doi:10.3389/fcvm.2021.765274)
Supplement: Supplementary file 3 [file Table_1.docx]

| **Supplementary Table 1. Output from the penalized Cox model.**  **HR** **Lower 95% CI** **Upper 95% CI**  **p** | | | | |
| --- | --- | --- | --- | --- |
| **NYHA III-IV** | **1.907** | **0.910** | **4.123** | **0.078** |
| **Sinus Rhythm** | **0.321** | **0.151** | **0.736** | **0.009** |
| **FT-derived RV GLS** | **1.053** | **0.995** | **1.104** | **0.060** |
| FT-derived LV GLS | 0.956 | 0.818 | 1.102 | 0.561 |
| **LGE** | **1.888** | **0.936** | **4.095** | **0.067** |
| LVEF (by CMR) | 0.996 | 0.924 | 1.075 | 0.922 |
| RVEF (by CMR) | 0.983 | 0.944 | 1.021 | 0.373 |
| LVEDVi (by CMR) | 1.006 | 0.998 | 1.014 | 0.120 |
| CMR = Cardiac Magnetic Resonance; EDVi = End Diastolic Volume indexed; EF = Ejection Fraction; FT = Feature Tracking; GLS = Global Longitudinal Strain; LGE = Late Gadolinium Enhancement; LV = Left Ventricle; NYHA = New York Heart Association; RV = Right Ventricle | | | | |

| **Supplementary table 2. Variability analysis. Intraclass Correlation Coefficients** | | | | | | |
| --- | --- | --- | --- | --- | --- | --- |
|  | **Intraobserver**  **Variability** |  | **Interobserver**  **Variability** |  | **Intercenter**  **Variability** |  |
|  | **ICC (95% CI)** |  | **ICC (95% CI)** |  | **ICC (95% CI)** |  |
| **Strain LV 2D** |  |  |  |  |  |  |
| LV peak GRS, % | 0.88 (0.79-0.94) |  | 0.92 (0.86-0.96) |  | 0.82 (0.59-0.93) |  |
| LV peak GCR, % | 0.76 (0.56-0.86) |  | 0.96 (0.92-0.98) |  | 0.95 (0.87-0.98) |  |
| LV peak GLS, % | 0.95 (0.91-0.97) |  | 0.96 (0.93-0.98) |  | 0.97 (0.93-0.99) |  |
| **Strain RV 2D** |  |  |  |  |  |  |
| RV peak GRS, % | 0.74 (0.54-0.86) |  | 0.94 (0.9-0.97) |  | 0.72 (0.40-0.88) |  |
| RV peak GCS, % | 0.81 (0.65-0.89) |  | 0.90 (0.81-0.94) |  | 0.67 (0.33-0.86) |  |
| RV peak GLS, % | 0.91 (0.83-0.95) |  | 088 (0.79-0.93) |  | 0.82 (0.59-0.93) |  |
| **RVEF, %** | 0.95 (0.92-0.96) |  | 0.92 (0.85-0.94) |  | 0.8 (0.75-0.84) |  |
| CI = confidence interval; EF = ejection fraction; GCS = global circumferential strain; GLS = global longitudinal strain; GRS = global radial strain; CI = confidence interval; ICC = intraclass correlation coefficient; LV = left ventricle; RV = right ventricle. | | | | | | |
